# First report of Iranian National nosocomial infectionsurveillance system software

**DOI:** 10.1186/1753-6561-5-S6-P231

**Published:** 2011-06-29

**Authors:** M Askarian, H Mahmoudi, O Assadian

**Affiliations:** 1 Community Medicine, Shiraz University of Medical Sciences, Shiraz, Iran, Islamic Republic Of; 2 University Hospital Vienna, Clinical Institute for Hygiene and Medical Microbiology Medical University Vienna, Vienna, Austria

## Introduction / objectives

Nosocomial infections are one of the most important health problems particularly in developing countries, because these infections cause high mortality and morbidity and have many economic loss consequences. To date however most surveillance studies have been conducted in most of developed countries, only a very few have been accomplished in Iran. The aim of this study is to determine the incidence of nosocomial infections in a big university hospital of Shiraz.

## Methods

The study was conducted prospectively through six months from 21^st^ March up to 22^nd^ September 2006 in a 374 bed educational hospital. All patients admitted during this period were included in the study and examined everyday for detecting four types of nosocomial infections: surgical site infection, urinary tract infection, pneumonia and blood stream infections. Centres for Disease Control and Prevention National Nosocomial Infection Surveillance system criteria were applied.

## Results

4013 patients were admitted in hospital. Overall infection rate was 2.45% and UTI, SSI, BSI and pneumonia rates were 1.07%, 0.72%, 0.32% and 0.32% respectively.

## Conclusion

To reduce nosocomial infections the first step is organizing a standard surveillance system, accurate and on time diagnosis of these infections and implying the infection preventing and control programs.

## Disclosure of interest

None declared.

